# Construction of novel phycocyanin conjugated chitosan–TiO_2_–Fe_3_O_4_ nanoparticles for *Lactobacillus* retainment and application in cyclic batch fermentation

**DOI:** 10.3389/fbioe.2026.1856231

**Published:** 2026-08-05

**Authors:** Varshashree Rajendran, Ravikumar Rajarathinam, S. Sudheer Khan, Prakash Kumar Sarangi, Vaibhav Srivastava

**Affiliations:** 1 Department of Biotechnology, Centre for Bioenergy and Bioproducts, Vel Tech Rangarajan Dr Sagunthala R&D Institute of Science and Technology, Chennai, Tamil Nadu, India; 2 Department of Oral Medicine and Radiology, Saveetha Dental College and Hospital, Saveetha Institute of Medical and Technical Sciences (SIMATS), Chennai, Tamil Nadu, India; 3 College of Agriculture, Central Agricultural University, Imphal, India; 4 Division of Glycoscience, Department of Chemistry, CBH School, Royal Institute of Technology (KTH), AlbaNova University Center, Stockholm, Sweden

**Keywords:** cell separation, Contois model, *Lactobacillus*, phycocyanin conjugation, TiO_2_–Fe_3_O_4_ nanoparticles

## Abstract

**Introduction:**

Many industries struggle to maintain culture during the repetitive batch fermentation process and also involve various downstream processing steps to separate microbial cells from the product. Hence, our study was focused on synthesising a novel biomaterial, a protein-conjugated magnetic nanoparticle, for *Lactobacillus* sp. retention and reuse and to reduce the process of cell-membrane filtration.

**Methods:**

Experimental studies were conducted to synthesise chitosan-coated phycocyanin-conjugated TiO_2_-doped Fe_3_O_4_ magnetic nanoparticles (Pc–Ch–TiO_2_–Fe_3_O_4_ NPs) for cell adhesion, and their potential was analytically characterised using FESEM, EDX, XRD, FT-IR, TGA, XPS, and VSM.

**Results:**

Cell separation efficiency, lactic acid yield, and kinetics were evaluated. *Lactobacillus* sp. were well-separated at an optimum concentration of 40 mg/100 mL using Pc–Ch–TiO_2_–Fe_3_O_4_ NPs, with a cell separation efficiency of 71.69%. The consistency of the Pc–Ch–TiO_2_–Fe_3_O_4_ NPs was again tested in a 5-L lab fermentor, leading to a lactic acid concentration of 23.65 g L^-1^ after 48 h in the third cycle. These findings confirm the method’s suitability for separating *Lactobacillus* sp. and highlight the strain’s robust fermentative performance across three consecutive batch cycles. Kinetic studies comprised a linear fit of the Contois model with μmax (h-1) = 0.47 and Ks = 3.855 in the initial cycle.

**Discussion:**

The investigation found that Pc–Ch–TiO_2_–Fe_3_O_4_ nanoparticles may effectively separate and retain *Lactobacillus* sp. in batch fermentation systems. In addition to enabling efficient magnetic cell recovery, the nanoparticles help to improve downstream processing by increasing the purity of lactic acid. This method is a viable alternative to traditional separation methods, enabling sustainable and cost-effective industrial bioprocessing.

## 1 Introduction

The essential benefits of probiotics can address the majority of our crucial nutritional and therapeutic supplement needs. By 2028, the probiotic industry is anticipated to grow to 95.25 billion dollars (Abedin et al., 2024). Lactic acid and poly(lactic acid) are widely known carboxylic acids found in nature and are frequently used organic acids in the food, pharmaceutical, textile, and packaging sectors (Castillo Martinez et al., 2013; John et al., 2009). The most prevalent lactic acid-producing bacteria are anaerobic to aerotolerant cocci or rods, Gram-positive, non-spore-forming, non-motile, catalase-negative, and cytochrome-free, which generate lactic acid and are resistant to acid, the primary byproduct of carbohydrate fermentation (Teneva-Angelova et al., 2018). A taxonomy review of the lactic acid producing bacteria in 2020 divided over 300 species *Lactobacillaceae* family, which consists of 31 genus and 23 novel species, genus to include microbes that were previously classified as species of *Lactobacillus* (Zhang et al., 2022). Lactic acid-producing bacterial genera have been utilized to produce fermented foods. The main lactic acid-producing bacteria in starter cultures used to produce fermented dairy products include Leuconostoc, Bifidobacterium, *Lactobacillus*, *Streptococcus*, and *Lactococcus lactis* (Karama et al., 2021).

Microorganisms are indispensable for human health and have substantial industrial utility. As a result, the fed-batch cultivation approach, which is used to achieve high cell densities in microbial strains commonly modified for the overexpression of recombinant or natural proteins, has emerged as a cornerstone methodology in biotechnology (Hewitt and Nienow, 2007). Scaling up a fermentation system involves more than just increasing the culture and vessel volume. Engineering concerns play a significant role. Unexpectedly, large-scale processes do not outperform small-scale laboratory processes (Enfors et al., 2001). Many industries were struggling to filter microorganisms from the lactic acid produced during fermentation. A continuous batch cell-recycle fermentation system for the production of lactic acid by *Lactobacillus paracasei* using polyvinylidene fluoride (PVDF) microfiltration (Xu et al., 2006). The main challenge in commercialising surfactin is its recovery and purification from complex fermentation broths. Downstream processing in biotechnological processes can account for up to 60% of the overall production costs (Isa et al., 2008). Traditionally, cells are separated from the medium using a high-g-force centrifuge. Microfiltration using membranes has recently emerged as a potential option. Both polymeric and ceramic membranes have been utilised for this purpose (Lee and Chang, 1990). This paper explores the use of a ceramic membrane filter to separate cells from the soup and analyses flow resistance when filtering the microorganism-containing broth (Li, 1996).

We report this study for the first time as an innovative application of chitosan-coated phycocyanin-conjugated TiO_2_-doped Fe_3_O_4_ magnetic nanoparticles (Pc–Ch–TiO_2_–Fe_3_O_4_ NPs) for cell separation, offering significant potential to reduce both the upstream and downstream processes in industrial bioreactors. This innovative method is especially beneficial for consecutive cycles, where cell recovery and cell reuse are paramount. In summary, this magnetic nanoparticle-based strategy provides a promising and streamlined solution for both the recovery of valuable microbial cells and their reuse in batch processes in industrial applications.

## 2 Materials and methods

### 2.1 Materials

FeCl_2_·4H_2_O (95% purity), FeCl_3_ 6H_2_O, chitosan, NaOH, titanium dioxide (97% purity), and glacial acetic acid (99.95% purity) were procured from Merck Company. Foetal bovine serum (FBS) 99% purity, penicillin, streptomycin, 3-(4,5-dimethyl-2-thiazolyl)-2,5-diphenyl-2H-tetrazolium bromide (MTT) (99.8% purity), and dimethyl sulfoxide (DMSO) (99% purity) were purchased from Sigma-Aldrich. All chemicals and reagents were used as supplied without any additional purification. *Spirulina (Arthrospira platensis)* was purchased from Ecolive Spirulina, Gobichettipalayam, Erode.

### 2.2 Synthesis of Ch–TiO_2_–Fe_3_O_4_ NPs

Iron oxide nanoparticles were synthesised by chemical co-precipitation. Iron (III) chloride hexahydrate (FeCl_3_·6H_2_O), 1.46 g, was dispersed in 100 mL of distilled water, followed by the addition of 0.54 g of iron (II) chloride tetrahydrate (FeCl_2_·4H_2_O) to the solution at 80 °C under continuous magnetic stirring. The Fe^3+^ and Fe^2+^ ions served as precursors for Fe_3_O_4_ nanoparticles. Titanium dioxide (0.2 g) was added as a dopant to the solution, denoted as Solution A (Shabani et al., 2016). In addition, 0.15 g of chitosan was mixed in 6 mL of 0.1% acetic acid and designated as solution B. Solution B was then mixed with solution A under continuous stirring at 500 rpm. After the co-precipitation method, 100 mL of 3 M NaOH solution (pH 10–13) was gradually added to the mixed solution at 80 °C while stirring. The alkaline condition facilitated the co-precipitation of Ch–TiO_2_–Fe_3_O_4_ nanoparticles, evidenced by the immediate formation of a black precipitate, which was continuously stirred at 500 rpm for 5 h. The precipitated solution was filtered using filter paper, washed thoroughly with distilled water to remove impurities, and dried at 50 °C. Dried particles were calcined at 600 °C in a muffle furnace for 8 h (Nair et al., 2023).

### 2.3 Extraction and purification of phycocyanin from *Arthrospira platensis*

*Arthrospira platensis*, used for extraction and purification, was purchased from Ecolive Spirulina, Gobichettipalayam, Erode. Initially, a specific volume of BG-11 medium was sterilised, and algae were sub-cultured. Parameters such as pH 10–11, room temperature, and a 12:12 light–dark cycle were maintained in the culture chamber throughout the experiment. The culture was harvested after 21–28 days and centrifuged at 5,000 rpm for 10 min at 4 °C. A total of 5 g of the collected biomass pellet was suspended in 50 mL of 0.1 M sodium phosphate buffer (pH 7) (Yucetepe et al., 2019). The cell disruption method was carried out using a bath sonicator operated at 50 kHz and 50 °C for 30 min, facilitating the release of phycocyanin into the buffer. The process could also be performed by sonicating on ice in short intervals or by homogenising at high speed. The sonicated suspension was then centrifuged at 10,000 rpm for 15 min–20 min to remove cellular debris, and the supernatant was collected and preserved at 4 °C for further analysis (Horváth et al., 2013).

For purification, ammonium sulphate was added to the crude extract to achieve 60% saturation, and the mixture was stirred for several minutes to promote protein precipitation. The solution was then stored at 4 °C overnight to allow complete precipitation of phycocyanin. The precipitated mixture was centrifuged at 10,000 rpm for 20 min at 4 °C, and the pellet was resuspended in an appropriate volume of sodium phosphate buffer. The dissolved fraction was transferred into dialysis tubing with a molecular weight cutoff of 12 kDa and dialysed against distilled water at 4 °C to remove residual ammonium sulphate. The purity and concentration of phycocyanin were determined by measuring the absorbance at 620 nm with a UV–Visible spectrophotometer. Finally, the purified extract was lyophilised to obtain freeze-dried phycocyanin from *Spirulina*. The quantity and purity of phycocyanin were evaluated by Equations 1, 2 (Nowruzi and Zakerfirouzabad, 2024) from measurements of the UV spectra.

$$\text{Extraction phycocyanin yield }\left(\%\right)=\frac{\text{Weight}\;\text{of}\;\text{extracted}\;\text{phycocyanin}}{\text{Dry}\;\text{biomass}\;\text{weight}}\;\times \;100.$$ (1)

$$\text{Phycocyanin ratio}=\frac{\text{Absorbance}\;\text{of}\;620\;\text{nm}}{\text{Absorbance}\;\text{of}\;280\;\text{nm}}.$$ (2)

### 2.4 Binding phycocyanin to Ch–TiO_2_–Fe_3_O_4_ NPs

A solution containing 50 μg/mL of iron oxide nanoparticles and 5% of glutaraldehyde was prepared and incubated for 45 min at 28 °C. Glutaraldehyde was used as a binding agent. It acts as a crosslinking agent, immobilising and linking proteins and enzymes. Glutaraldehyde has two functional groups that bind to chitosan’s amino groups. Excess glutaraldehyde was eliminated, and the sample was rinsed thrice with 0.01 M phosphate-buffered saline (PBS) at pH 7–10. To facilitate the conjugation of phycocyanin and Ch–TiO_2_–Fe_3_O_4_ NPs, a concentration of 500 μg/mL of phycocyanin was prepared and added to the nanoparticle solution. The mixture was then incubated overnight at 4 °C. The conjugation efficiency was determined by measuring the absorbance of the initial phycocyanin concentration and the unbound phycocyanin concentration in the supernatant at 620 nm using a UV–Visible spectrophotometer, according to Equation 3.

$$\text{Conjugation efficiency }\left(\%\right)=\frac{\text{Initial}\;\text{absorbance}\;\text{at}\;620\;\text{nm}‐\text{Unbound}\;\text{absorbance}\;\text{at}\;620\;\text{nm}\;}{\text{Initial}\;\text{absorbance}\;\text{at}\;620\;\text{nm}}\;\times \;100.$$ (3)

**FIGURE 1:**
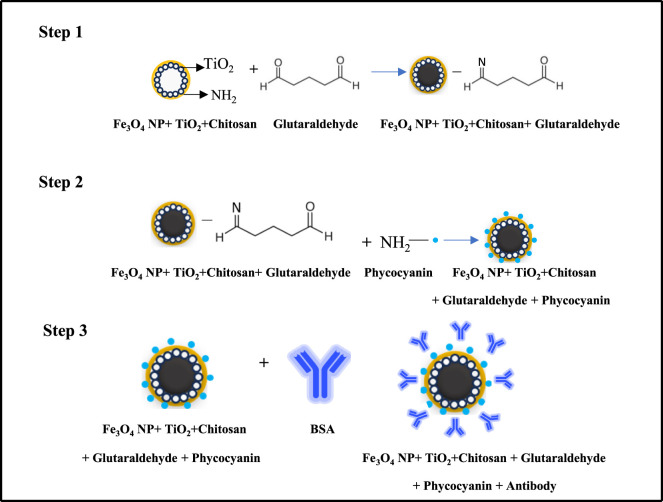
Step-by-step synthesis of Pc–Ch–TiO_2_–Fe_3_O_4_ NPs.

The conjugation efficiency of phycocyanin onto Ch–TiO_2_–Fe_3_O_4_ nanoparticles was found to be 90.98%. The conjugated nanoparticles were rinsed thrice with PBS and then incubated with .5% bovine serum albumin (BSA) to eliminate any non-specific binding on the nanoparticle surface (Huy et al., 2014). Figure 1 shows the process of conjugation of Pc–Ch–TiO_2_–Fe_3_O_4_ NPs.

### 2.5 MTT assay for the Vero cell line

Vero cells were sourced from the National Centre for Cell Sciences (NCCS), Pune, India, and cultured in a medium containing 10% FBS, penicillin (100 IU/mL), and streptomycin (100 μg/mL). The cells were sustained at 37 °C in an incubator with 5% CO_2_ until a confluent monolayer developed. The adherent cells were detached with trypsin, and the suspension thus obtained was adjusted to a density of 1.0 × 10^5^ cells/mL in a medium containing 10% FBS. Aliquots of 100 μL (equivalent to 1 × 10^4^ cells/well) were dispensed into 96-well plates and incubated for 24 h to facilitate partial monolayer formation. After incubation, the spent medium was eliminated, and the wells were rinsed once with fresh medium. Subsequently, 100 μL of Pc–Ch–TiO_2_–Fe_3_O_4_ nanoparticle suspensions at various concentrations (20, 40, 60, 80, and 100 μg/mL) were dispersed in the wells and incubated again at 37 °C under 5% CO_2_ for 24 h. Following treatment, the nanoparticle suspensions were removed, and 20 μL of MTT solution (2 mg/mL in PBS) was introduced to each well. The plates were then incubated for 4 h at 37 °C in a 5% CO_2_ atmosphere to allow the formation of formazan. The supernatant was carefully discarded, and 100 μL of DMSO was added to dissolve the formazan crystals. The absorbance was measured at 570 nm using a microplate reader. The percentage of viable cells was evaluated using Equation 4.

$$\%\text{ of viability}=\frac{\text{Sample}\;\text{absorbance}}{\text{Control}\;\text{absorbance}}\;\times \;100.$$ (4)

### 2.6 Batch experimental and optimization studies of Pc–Ch–TiO_2_–Fe_3_O_4_ NPs in flask and fermentor

Batch experiments were conducted to optimise the concentration of Pc–Ch–TiO_2_–Fe_3_O_4_ NPs for cell separation, focussing on separation efficiency. To evaluate the specificity and effectiveness of the nanoparticle-assisted separation system, appropriate control experiments were also performed. A control experiment without nanoparticles was conducted using conventional centrifugation at approximately 8,000 rpm and 4 °C for 10 min to compare the bacterial separation efficiency and cell recovery. The cell recovery Equation 5 was used to determine cell separation efficiency.

$$\text{Cell recovery or cell separation }\%=\frac{\text{Initial}\;\text{absorbance}\;\text{at}\;600\;\text{nm}}{\text{Final}\;\text{absorbance}\;\text{at}\;600\;\text{nm}}\;\times \;100.$$ (5)

In addition, unconjugated Ch–TiO_2_–Fe_3_O_4_ nanoparticles (without phycocyanin conjugation) were used as a control to assess the specific contribution of phycocyanin in bacterial binding and separation. *Lactobacillus* sp. (accession number: PQ836145) culture was collected from Prathista Industries, Limited, Telangana. Initially, *Lactobacillus* sp. were cultivated in five separate 250 mL conical flasks with a working volume of 100 mL using MRS broth medium, with an inoculum of 2.5 mL, in an incubator shaker maintained at 37 °C for 48 h at 50 rpm. After incubation, the initial OD of the culture medium was measured at 600 nm using UV spectrophotometry after 48 h. Different concentrations of Pc–Ch–TiO_2_–Fe_3_O_4_ NPs (10 mg/100 mL–50 mg/100 mL) were added to the above flasks, and the entire culture medium was allowed to remain undisturbed for 4 h for cell binding. From each conical flask, the Pc–Ch–TiO_2_–Fe_3_O_4_ NPs were separated using a neodymium magnet, and the OD value (final) was recorded to estimate the bacterial cell separation using Equation 5 (Delahaye et al., 2015).

Furthermore, the viability of the separated bacterial cells was assessed by colony-forming unit (CFU) analysis on MRS agar plates to determine the effect of nanoparticle-assisted separation on bacterial survival. In addition, the separated Pc–Ch–TiO_2_–Fe_3_O_4_ NPs with the cells were further observed under a fluorescent microscope, LYNX, India, and FESEM analysis was carried out to examine the binding interaction between the bacterial cells and Pc–Ch–TiO_2_–Fe_3_O_4_ NPs. To check the consistency of the optimised concentration of the Pc–Ch–TiO_2_–Fe_3_O_4_ NPs, the experiments were repeated using 5-L *in situ* sterilisation-type lab fermenter 3i, Borg scientific, India, with the working volume of 1 L using deMan, Rogosa, and Sharpe (MRS) broth medium operated at 37 °C temperature with the agitation speed of 50 rpm (Jangra et al., 2015). The pH of the fermentation medium was periodically monitored and maintained within the desired range using sterile 1M NaOH and 1 M HCl (acid/base) solutions when necessary. Dissolved oxygen (DO) levels were continuously monitored with an *in situ* DO probe and maintained above 30% saturation throughout the fermentation process to ensure sufficient oxygen for microbial growth and metabolite production. The samples were collected at 0-h intervals up to 48 h for analysis. All experiments were performed in triplicate, and the results are expressed as the mean ± standard deviation (SD). A Rushton turbine impeller with five blades was used for agitation.

In the first phase (initial cycle), without the addition of Pc–Ch–TiO_2_–Fe_3_O_4_ NPs, the fermentation process to convert the dextrose present in the MRS medium into lactic acid was allowed to proceed for 48 h. Parameters such as biomass concentration, cell dry weight, substrate concentration, lactic acid production, and pH were monitored and recorded at regular time intervals. Growth kinetics of *Lactobacillus* sp. were determined from OD measurements at different time intervals. After 48 h, the cells were attached by adding the optimised concentration of the Pc–Ch–TiO_2_–Fe_3_O_4_ NPs to the medium using magnets at the outer walls of the fermentor. Later, in the second phase (cycle 1), the medium was removed from the vessel, and fresh medium was added to the same fermenter. Here, the magnets were detached from the fermentor vessel, thereby simultaneously allowing the cells to regrow and release the product, lactic acid. The regrowth was monitored and observed. The batch process was repeated for cycles 2 and 3 to identify the maximum cycle by measuring the growth kinetics, biomass concentration, cell dry weight, substrate concentration, lactic acid production, and pH. The biomass concentration was measured at 600 nm using UV spectrophotometry. A measure of 10 mL of *Lactobacillus* sp. culture broth was taken into a 100-mL conical flask and titrated with 0.1 N sodium hydroxide solution. Phenolphthalein was used as an indicator, and the observed titre value was used to determine the percentage of lactic acid. Lactic acid (LA g L^-1^) concentration was determined using Equation 6 at different time intervals (Chelli et al., 2017).

$$\text{Lactic acid }\%=\text{Titer value }\left(\text{NaOH}\right)\;\times \;0.00908\;\times \;100.$$ (6)

The consumption of dextrose (substrate concentration, S g L-1) was determined by measuring the reducing sugars using the NS method as described by Miller (1959). pH was measured using a Mettler Toledo pH metre (Switzerland). The cell dry weight (X g L^-1^) was determined based on the initial and final filter weights of the cells (Rezvani et al., 2017).

### 2.7 Kinetic models

A model independent of substrates and growth pattern utilized the Contois, Monod, logistic, and Gompertz equations to characterise microbial cell growth during a fermentation process over four cycles and was thoroughly analysed. Contois is an unstructured kinetic model based on substrate and biomass concentration. To determine the kinetic constants for the regrowth of *Lactobacillus* sp., the Contois model Equation 7 was used for the initial cycle (cycle 1), cycle 2, and cycle 3. The effectiveness of the Pc–Ch–TiO_2_–Fe_3_O_4_ NPs for cell separation in a repetitive batch process was also analysed.

$$µ=µ\max\frac{S\;}{K_{s}X+S}.$$ (7)

**FIGURE 2:**
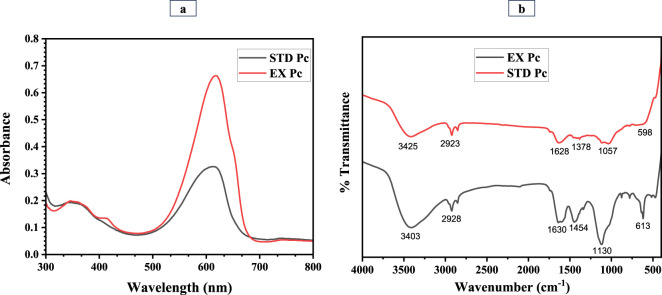
**(a)** UV–Visible absorption spectra of STD Pc (standard phycocyanin) and EX Pc (extracted phycocyanin), **(b)** FT‐IR spectra of STD Pc (standard phycocyanin) and EX Pc (extracted phycocyanin).

**FIGURE 3:**
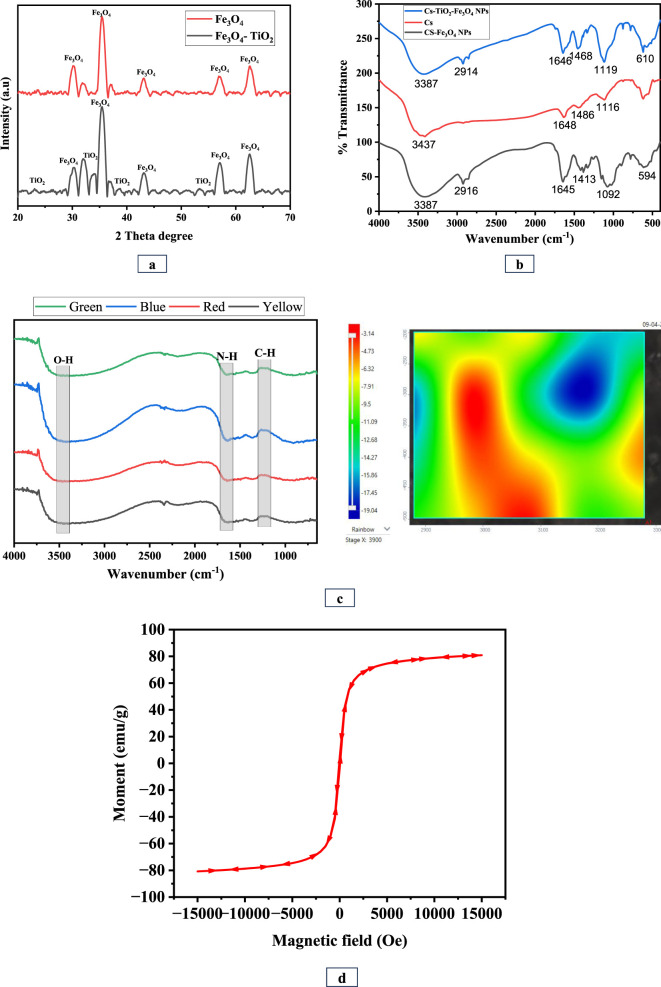
**(a)** XRD patterns of Ch–TiO_2_–Fe_3_O_4_ NPs. **(b)** FT‐IR analysis of Cs–TiO_2_–Fe_3_O_4_ NPs (chitosan‐conjugated TiO_2_‐doped Fe_3_O_4_ nanoparticles, Cs = chitosan, Cs–Fe_3_O_4_ NPs, chitosan‐conjugated Fe_3_O_4_ naoparticles). **(c)** Micro‐FT‐IR analysis of Pc–Ch–TiO_2_–Fe_3_O_4_ NPs. **(d)** Hysteresis loop of Ch–TiO_2_–Fe_3_O_4_ NPs.

Here, µ = specific growth rate (h^-1^), µ_max_ = maximum specific growth rate (h^-1^), S = substrate concentration (g L^-1^), X = biomass concentration (g L^-1^), and K_s_ = half-saturation constant. Table 1 and Figures 2, 3 show the linear fits using the **C**ontois model for each cycle, respectively.

$$\frac{\text{dX}}{\text{dt}}=µX.$$ (8)

**TABLE 1:** Kinetic constants linearised fit of *Lactobacillus* sp. for the four studied cycles.

Kinetic models	Cycles	µ_max_ (h^-1^)	K_S_ (g L^-1^)	R^2^
Contois model	Initial cycle	0.464	3.85	0.8633
	Cycle 1	0.472	3.788	0.8732
	Cycle 2	0.496	3.98	0.8794
	Cycle 3	0.651	7.53	0.9303
Monod model	Initial cycle	0.401	14.63	0.816
	Cycle 1	0.415	14.31	0.821
	Cycle 2	0.446	15.4	0.8315
	Cycle 3	1.05	51.73	0.8713
Logistic model	Initial cycle	0.3293	*NA*	0.3568
	Cycle 1	0.5582	*NA*	0.8011
	Cycle 2	0.3983	*NA*	0.7496
	Cycle 3	0.638	*NA*	0.8549
Gompertz model	Initial cycle	0.3313	*NA*	0.4839
	Cycle 1	0.3486	*NA*	0.5255
	Cycle 2	0.3158	*NA*	0.4933
	Cycle 3	0.3615	*NA*	0.4948

Equation 8 shows that the cell concentration increases exponentially with time as long as the specific growth rate (μ) remains constant, representing the Malthusian growth (Malthus law) of *Lactobacillus* sp. in a batch culture.

$$\mu =\frac{\ln\;\frac{X}{X_{0}}\;}{t‐t_{0}}.$$ (9)

Integration of Equation 7 with the suitable initial condition (X = X0 at t = t0) yielded Equation 9, which represents μ.

The Monod model describes the relationship between the specific growth rate and the substrate utilisation rate in a bioreactor.

$$µ=µ\max\frac{S\;}{K_{s}+S}.$$ (10)

Integration of Equation 10 using the suitable initial condition (X = X_0_ at t = t_0_) yielded Equation 11, which represents μ.

$$\ln\left(\frac{X}{X_{0}}\right)=µ\max\frac{S\;}{K_{s}+S}\;t.$$ (11)

The logistics model describes the growth pattern in a bioreactor**.**

$$\frac{\text{dX}}{\text{dt}}={µ}_{\max}X\;\left(1‐\frac{X}{X_{\max}}\right).$$ (12)

The integration of Equation 12 yields the following:

$$X=\frac{X_{0}e^{{µ}_{m}t}}{\left\{1‐\left(X_{0}/X_{m}\right)\left(1‐e^{{µ}_{m}t}\right)\right\}}.$$ (13)

The Gompertz growth model is commonly used for the growth pattern in fermentation studies.

$$X=\text{Xmax}\exp\left[‐\;\exp\left(‐k\left(t‐\text{ti}\right)\right)\right].$$ (14)

The integration of Equation 14 yields the following:

$$‐\ln\left(\frac{X}{X_{\max}}\right)=‐\text{ kt}+\text{kti}.$$ (15)

### 2.8 Analytical characterisation

Numerous analytical characterisations were used to evaluate the nanoparticles and phycocyanin, including a UV–Vis spectrophotometer and field emission scanning electron microscopy (FESEM) with energy dispersive X-ray spectroscopy (EDX) (Carl Zeiss Sigma 360 Gemini 1, Germany). X-ray diffraction (XRD) (BRUKER, Germany) and Fourier transform infrared spectroscopy (FT-IR) (PerkinElmer Spectrum Two FT-IR instrument and Micro FT-IR—Nicolet iS50 FT-IR Spectrometer) were also used for further examination. X-ray photoelectron spectroscopy (XPS) from Thermo Fisher Scientific, United States, was used to analyse the chemical state of the nanoparticles. Thermogravimetric analysis (TGA) on a NETZSCH STA 449 F3 was performed, and a Vibrating Sample Magnetometer (VSM) from Lakeshore VSM 7410S, United States of America, was used to validate the magnetic properties of Pc–Ch–TiO_2_–Fe_3_O_4_ NPs. Fluorescent microscopy was used to validate the adhesion between Pc–Ch–TiO_2_–Fe_3_O_4_ NPs and *Lactobacillus* sp.

## 3 Results

### 3.1 Absorption spectrum of phycocyanin

A UV–Visible spectrophotometer was used to measure the standard phycocyanin and purified phycocyanin using a dialysis membrane with a molecular size of 50 due to the molecular size of the phycocyanin. In comparison, extracted phycocyanin has high purity and concentration. A sharp visible peak at 620 nm confirms the presence of tetrapyrrole in the region, and it may differ from 610 nm to 620 nm, as shown in Figure 2a. The purity index of the extracted phycocyanin was calculated using Equation 2 (Narindri et al., 2025) and was found to be 1.13 (food grade), and 10.8% extraction yield was found in the extracted phycocyanin. The hump at 345 nm indicates the presence of phycobilin chromophore and carotenoids.

### 3.2 FT-IR analysis of phycocyanin

The FT-IR spectra of the extracted and standard phycocyanin nanoparticles were recorded over 4,000 cm^-1^ to 400 cm^-1^, as shown in Figure 2b. The differences between purified and standard phycocyanin are evident in the peak positions and intensities of their FT-IR spectra. Peaks appear similar, and the intensity changes in the FT-IR analysis are observed for both phycocyanins. Spectral analysis was used to determine the functional groups in the extracted and standard phycocyanin; the peaks at 3,403 cm^-1^ and 3,425 cm^-1^ confirm the O–H stretching band. The rough peaks at 2,928 and 2,923 cm^-1^ indicate C–H stretching and the presence of alkane in phycocyanin due to the sodium phosphate buffer used in the extraction process. The peaks at 1,630 and 1,628 cm^-1^ correspond to the tetrapyrrole chain of C_4_H_5_N.

Furthermore, the peaks at 1,130 and 1,057 cm^-1^ illustrate C–F bands, indicating the presence of a strong fluorescent compound in phycocyanin. In addition, peaks between 900 cm^-1^ and 590 cm^-1^ indicate the presence of aromatic groups in phycocyanin. Although minor variations in the peak intensity were observed between purified and standard phycocyanin, the overall spectral similarity confirms that the extraction and purification process preserved the key structural features of phycocyanin. Therefore, the FT-IR results validate the successful extraction and purification of phycocyanin with retained functional groups, making it suitable for subsequent conjugation and cell separation applications.

### 3.3 XRD analysis of Ch–TiO_2_–Fe_3_O_4_ NPs

The crystalline phase composition and structural characteristics of the synthesised Ch–TiO_2_–Fe_3_O_4_ nanoparticles were systematically analysed using powder X-ray diffraction (XRD) analysis utilizing Cu Kα radiation with a wavelength of λ = 1.54 Å. The XRD pattern obtained, presented in Figure 3a, exhibited well-resolved and distinct diffraction peaks attributable to both the cubic inverse spinel structure of magnetite (Fe_3_O_4_, JCPDS card No. 75–0449) and the characteristic crystalline phases of titanium dioxide (TiO_2_, JCPDS card No. 21–1272). Unambiguous peak assignment provides compelling evidence for the successful co-precipitation and integration of the magnetic Fe_3_O_4_ dopant within the TiO_2_–chitosan matrix. Specifically, the Fe_3_O_4_ phase was identified by its signature Bragg reflections at 2θ values of 32.0°, 35.44°, 57.0°, 62.56°, and 71.44°, which correspond precisely to the high-symmetry (220), (311), (511), (440), and (622) crystallographic planes, respectively. These peak positions are hallmarks of magnetite nanoparticles and confirm the retention of the cubic spinel framework during synthesis. Concurrently, the TiO_2_ component exhibited distinct diffraction features: anatase-phase reflections at 24.8° (101), 38.65° (112), and 48.8° (200), along with rutile-phase peaks at 67.76° (301) and 74.28° (310). The coexistence of both anatase and rutile polymorphs is noteworthy, as this biphasic structure often enhances the photocatalytic performance through synergistic electronic interactions at phase boundaries. The pronounced sharpness, high intensity, and well-defined nature of all the observed peaks collectively indicate a high degree of crystallinity and minimal structural defects within the nanocomposite. To quantify the nanoscale dimensions, the crystallite size was determined via the Debye–Scherrer Equation 16.

$$D=\frac{K\lambda }{\beta \mathrm{Cos}\theta }.$$ (16)

Here, D = particle size, K = Scherrer constant (0.94), λ = X-ray wavelength (nm), β = peak width of the half maximum (FWHM), and θ = Bragg diffraction angle (rad). Applying this methodology to the most prominent (311) peak of Fe_3_O_4_ yielded an average crystallite size of 44.55 nm. This size is critical, as the properties of the dopant nanoparticles depend strongly on their dimensions. Nanoparticles in this range typically offer a high surface area-to-volume ratio, which is beneficial for applications such as adsorption and catalysis. The distinct peak positions and relative intensities in the diffractogram of the Ch–TiO_2_–Fe_3_O_4_ nanoparticles unequivocally confirm the formation of a face-centred cubic crystalline lattice, with phase purity and structural integrity preserved throughout the synthesis protocol. This nanoscale dimension is critically important, as the physicochemical properties of such dopant nanoparticles, including the magnetic susceptibility, photocatalytic efficiency, and dispersibility, are profoundly influenced by their size. Nanoparticles in this size range typically exhibit exceptionally high surface-to-volume ratios, which are particularly advantageous for applications in cell separation, cell adhesion, and environmental remediation, such as heavy-metal adsorption, organic pollutant degradation, and heterogeneous catalysis.

### 3.4 FT-IR analysis of Ch–TiO_2_–Fe_3_O_4_ NPs

The functional groups of the present substances can be identified using the FT-IR data. The FT-IR spectra of the produced nanoparticle were recorded on the 4,000 cm^-1^–400 cm^-1^ scale, as illustrated in Figure 3b. The analysis was used to determine the functional groups involved in the capping and durability of chitosan, Ch–Fe_3_O_4_ NPs, and Ch–TiO_2_–Fe_3_O_4_ NPs. The peaks at approximately 610 cm^-1^ and 594 cm^-1^ coincide with the Fe–O bond in Ch–TiO_2_–Fe_3_O_4_ NPs and Ch–Fe_3_O_4_ NPs. The peak at 594 cm^-1^ indicates the Ti–O bond and Fe–O bond in Ch–TiO_2_–Fe_3_O_4_ NPs. The spikes at 1,648 cm^-1^ correspond to the NH_2_ bonds for natural chitosan. The cell wall of *Lactobacillus* sp. is rich in negatively charged components, including teichoic acids and peptidoglycan. The protonated amino groups (–NH_3_^+^) of chitosan, as evidenced by FT-IR peaks in the amide region, exhibit strong electrostatic attraction to these negatively charged sites, facilitating rapid and stable attachment of nanoparticles to the cell surface. The TiO_2_–Fe_3_O_4_ core nanoparticles are surface-modified after conjugation with chitosan and phycocyanin. The peaks for Ch–Fe_3_O_4_ NPs and Ch–TiO_2_–Fe_3_O_4_ NPs blends appear at 1,645 cm^-1^ and 1,646 cm^-1^, respectively, due to chitosan conjugation to the magnetic nanoparticles. The peaks at 3,437 cm^-1^, 3,387 cm^-1^, and 3,387 cm^-1^ are attributed to O–H stretching in all samples; the peaks at 2,916, 2,899, and 2,914 cm^-1^ are attributed to C–H stretching. The spikes at 1,450, 1,477, and 1,391 cm^-1^ corresponded to the O–H bending of water. The presence of these functional groups confirms the effective surface modification and the formation of a stable organic coating on the nanoparticle surface. These functional moieties play a crucial role in facilitating interactions with microbial cells.

### 3.5 Micro FT-IR analysis of Pc–Ch–TiO_2_–Fe_3_O_4_ NPs

The functional groups of the present substances can be identified using the micro-FT-IR values obtained. The FT-IR spectra analysed for Pc–Ch–TiO_2_–Fe_3_O_4_ NPs were in the 4,000 cm^-1^–400 cm^-1^ wavelength (red shows high concentration of Fe, and blue shows low concentration of titanium), as illustrated in Figure 3c. The peaks at approximately 3,500 cm^-1^ are due to O–H stretching in water. The N–H stretching at 1,600 cm^-1^, corresponding to NH_2_ bonds, is observed for natural chitosan. The hump between 1,200 and 1,300 cm^-1^ shows a C–H stretch, indicating the presence of an alkane in phycocyanin. FT-IR analysis provides critical insights into the surface chemistry of Pc–Ch–TiO_2_–Fe_3_O_4_ nanoparticles and their interaction with *Lactobacillus* sp. The presence of broad O–H/N–H stretching bands (∼3,437 cm^-1^–3,387 cm^-1^) and amide bands (∼1,645 cm^-1^–1,646 cm^-1^) confirms the abundance of hydroxyl and amino groups derived from chitosan and phycocyanin on the nanoparticle surface. These functional groups play a key role in mediating interactions with bacterial cells. Overall, the micro-FT-IR spectrum confirms the successful integration of phycocyanin, chitosan, TiO_2_, and Fe_3_O_4_ within the nanoparticles. The observed O–H, N–H, and C–H vibrational bands, along with characteristic metal–oxygen signatures, collectively validate the presence of both organic functional groups and inorganic oxide components, indicating the effective formation of Pc–Ch-TiO_2_–Fe_3_O_4_ nanoparticles.

### 3.6 Magnetic property

The magnetic characteristics of the synthesised Ch–TiO_2_–Fe_3_O_4_ NPs were analysed to evaluate the magnetic behaviour. The VSM plot in Figure 3d exhibits a well-defined S-shaped hysteresis loop, confirming the superparamagnetic nature of the nanoparticles. The measured saturation magnetisation was approximately 80 emu/g, indicating strong magnetic responsiveness without residual magnetisation. The results obtained in the present study fall within this reported range, further validating the magnetic performance of the Ch–TiO_2_–Fe_3_O_4_ NPs.

### 3.7 Morphological characterisation and EDX elemental mapping

**FIGURE 4:**
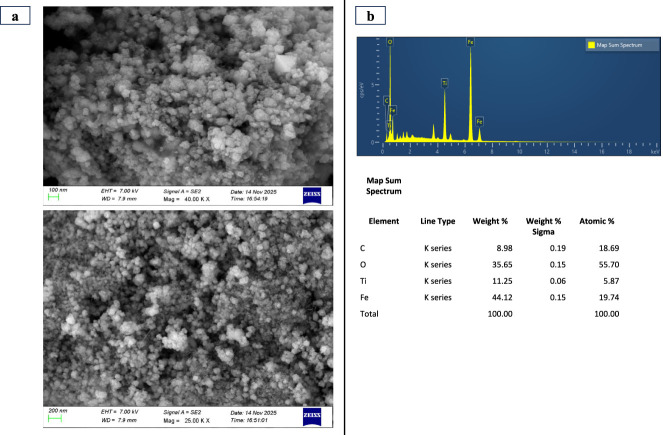
**(a)** Morphological appearance of Ch–TiO_2_–Fe_3_O_4_ NPs. **(b)** Elemental analysis of Ch–TiO_2_–Fe_3_O_4_ NPs.

The surface morphology, particle size distribution, and elemental composition of the as-synthesised Ch–TiO_2_–Fe_3_O_4_ nanoparticles were comprehensively characterised using field-emission scanning electron microscopy (FESEM) integrated with energy-dispersive X-ray (EDX) spectroscopy. High-resolution FESEM micrographs (Figure 4a) revealed a homogeneous distribution of quasi-spherical nanoparticles across the sampled regions, with evident agglomeration primarily driven by intermolecular van der Waals forces from the ferrimagnetic Fe_3_O_4_ cores. The nanoparticles are as small as possible for effective cell separation. The observed dense packing of variably sized nanoparticles within these aggregates indicates a polydisperse size distribution, a feature common in wet-chemical synthesis routes and contributing to enhanced colloidal stability. Surface roughness is observed in the FESEM image, which might enhance TiO_2_’s surface properties, such as adsorption, surface area, and chemical reactivity. The agglomeration of particles might be due to magnetic remanence between them. EDX ensures the elements’ composition present in Ch–TiO_2_–Fe_3_O_4_ NPs. The Fe peaks and table are visible in the EDX (Figure 4b), indicating the presence of Fe in the nanoparticles. Ti indicates the presence of Ti as a dopant. Additional components, such as carbon (C), indicate the presence of chitosan, and O shows the presence of oxides. The peaks at 0.25 and 6.5 keV represent Fe binding energies. The spike at 0.5 keV is assigned to O. The peaks at 0.25 and 4.5 keV are assigned to Ti. The spike at 0.2 keV shows C. The synthesised nanoparticles comprised iron, titanium, oxygen, and carbon at approximately 44.12%, 11.25%, 35.65%, and 8.98%, respectively.

### 3.8 Thermogravimetric analysis

**FIGURE 5:**
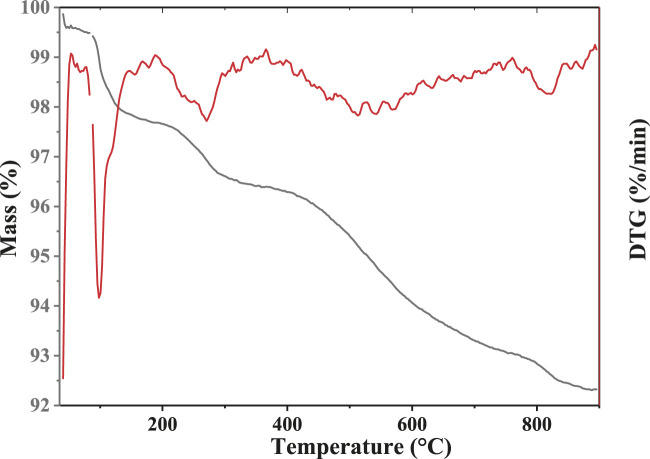
TGA and DTG measurement of the functionalised Pc–Ch–TiO_2_–Fe_3_O_4_ NPs.

Thermogravimetric analysis (TGA) and derivative thermogravimetric (DTG) curves of the functionalised Pc–Ch–TiO_2–_Fe_3_O_4_ NPs are presented in Figure 5. The TGA profile showed gradual mass loss over 30 °C–900 °C, indicating the thermal stability and compositional characteristics of the nanoparticles. An initial weight loss of 1.518% below ∼150 °C is attributed to the removal of physically adsorbed moisture and surface-bound volatile components. The second stage, with a mass loss of 1.461% between ∼150 °C and 350 °C, corresponds to the thermal degradation of organic components, notably chitosan and phycocyanin, demonstrating their efficient functionalisation on the nanoparticle surface. This stage represents the degradation of polymeric chains and protein molecules. Between 350 °C and 600 °C, there is a considerable weight loss of 1.925% due to the disintegration of organic residues and structural rearrangements inside the nanoparticles. This indicates a strong interaction between the organic covering and the inorganic core. At higher temperatures (∼600 °C–800 °C), a minor mass loss of 1.46% is attributed to the removal of the residual carbonaceous matter. Beyond this region, the mass remained almost constant, indicating the presence of thermally stable inorganic phases such as TiO_2_ and Fe_3_O_4_. The nanoparticles’ exceptional heat stability is demonstrated by their weight loss of approximately 6.3%. These findings are consistent with FT-IR observations, which showed distinctive O–H/N–H stretching at ∼3,400 cm^-1^, amide bands at 1,600 cm^-1^–1,650 cm^-1^, and Fe–O vibrations at 500 cm^-1^–700 cm^-1^, indicating the presence of chitosan, phycocyanin, and iron oxide. XRD measurements confirmed the crystalline structures of TiO_2_ and Fe_3_O_4_, with no substantial structural changes observed following functionalisation. These results confirm the effective synthesis of thermally stable, structurally robust, and functionally integrated Pc–Ch–TiO_2_–Fe_3_O_4_ nanoparticles appropriate for bioprocess applications.

### 3.9 XPS analysis

**FIGURE 6:**
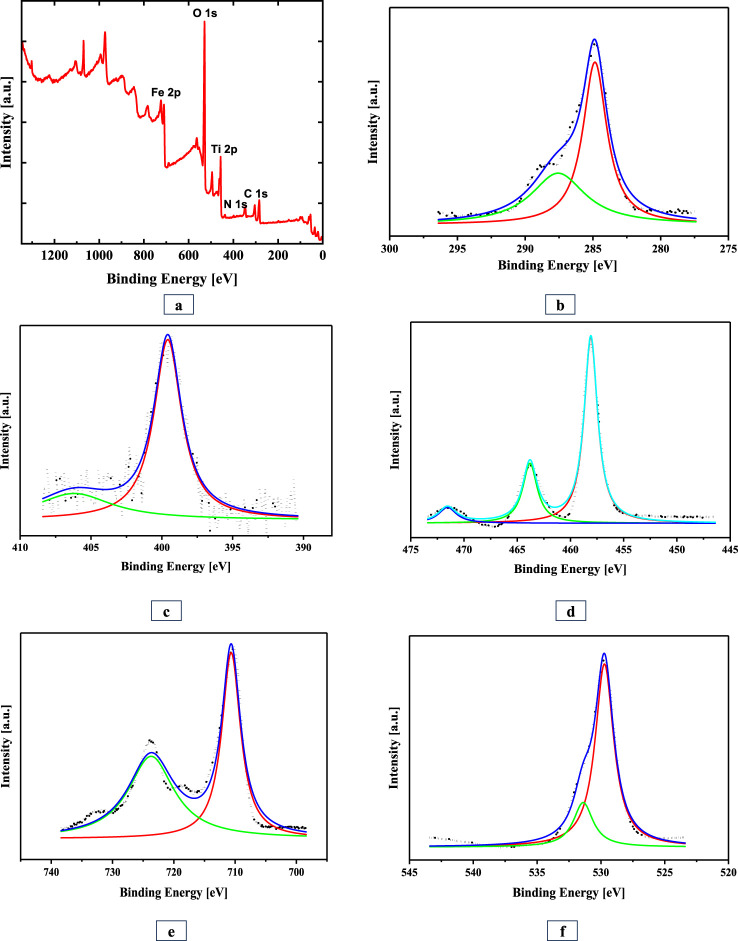
XPS spectra for Ch–TiO_2_–Fe_3_O_4_ NPs: **(a)** general experimental spectrum; **(b–f)** interpreted values for C, N, Ti, Fe, and O, respectively.

X-ray photoelectron spectroscopy was used to analyse the chemical state and elemental composition of the synthesised Pc–Ch–TiO_2_–Fe_3_O_4_ NPs, as shown in Figure 6. The Fe 2p spectrum was deconstructed into three main components. The Fe 2p_3_/_2_ signal at 710.5 eV corresponds to Fe^3+^, which is consistent with the oxidation state of Fe_3_O_4_. Between 723 eV–726.3 eV in the Fe 2p_1_/_2_ region, powerful satellite peaks caused by multiplet splitting may be detected. Satellite structures form from the interaction between the 2p core hole and the unpaired 3d electrons of Fe^3+^ (3d^5^ configuration). Fe^3+^ 3d orbitals contain five unpaired electrons, allowing for different spin configurations and satellite transitions. These satellite peaks indicate that Fe^3+^ is the dominant ligand in an octahedral environment. The O 1s core-level spectrum has two components: a prominent peak at 529.7 eV, corresponding to lattice O^2-^ linked to Fe and Ti in a metal–oxygen octahedral framework, where O 2p orbitals strongly hybridise with Fe/Ti 3d orbitals. TiO_2_–Fe_3_O_4_ exhibits a second peak at ∼530.0 eV, indicating oxygen vacancies or defects due to partial reduction or lattice distortion. These flaws alter the electrical structure by introducing donor-like states near the conduction band. The Ti 2p spectra have discrete peaks at 458.2 eV (Ti 2p_3_/_2_) and 463.9 eV (Ti 2p_1_/_2_), indicating mixed-valence Ti^3+^ and Ti^2+^ states created by ion bombardment or defect-induced reduction. Titanium oxides rely heavily on their 3d orbitals; decreased Ti^3+^/Ti^2+^ ratios have lower binding energies than stoichiometric Ti^4+^. These characteristics imply oxygen-deficient TiO_2_, which increases the electron mobility and surface reactivity. The N 1s spectrum shows two peaks at 400 eV and 399.5 eV, corresponding to the protonated amine (-NH_3_^+^) and amide/amine (-NH_2_) groups of chitosan on the nanoparticle surface. Chitosan’s nitrogen exhibits sp^3^ hybridisation, with lone-pair electrons in a non-bonding orbital, which contribute to modest chemical changes when interacting with metal oxide surfaces via hydrogen bonding or coordination. The C 1s spectrum is divided into two parts: aliphatic carbon (C–C/C–H) at 284.7 eV, which represents the organic backbone of chitosan, and C–O/C–N groups at 286.7 eV, which correspond to the functional groups of phycocyanin attached to the nanoparticle surface. These higher-binding-energy peaks are caused by electron-withdrawing oxygen and nitrogen atoms, which redistribute electron density on the carbon atom, thereby shifting the peak.

### 3.10 Cytotoxic activity

**FIGURE 7:**
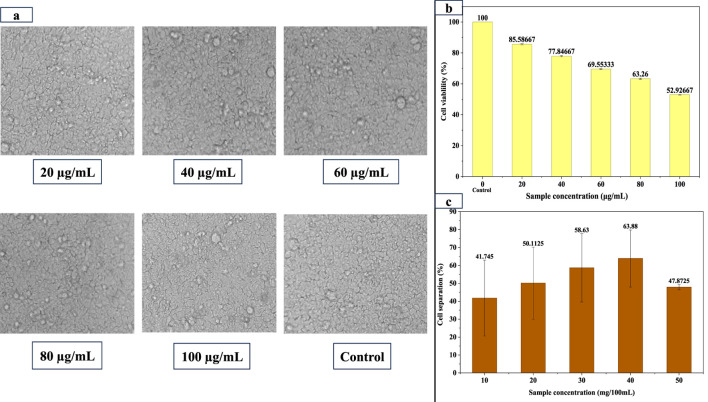
**(a)** MTT assay for the biocompatibility of Pc–Ch–TiO_2_–Fe_3_O_4_ NPs. **(b)** Anti‐proliferation effects of Pc–Ch–TiO_2_–Fe_3_O_4_ NPs. **(c)** Optimisation of cell separation.

The cytotoxic effects of the Pc–Ch–TiO_2_–Fe_3_O_4_ NPs under study were assessed in the Vero cell line. Due to the chemotherapeutic drugs, nephrotoxicity endures as an awful side effect that inhibits the treatment effectiveness. Synthesised nanoparticles were evaluated at various concentrations (20, 40, 60, 80, and 100 μg/mL). At 20 μg/mL, it shows high biocompatibility, with Vero cells showing 85.59% ± 0.45% cell viability. The cytotoxicity assay showed that the tested sample reduced cell viability in a concentration-dependent manner. The IC_50_ value was approximately 105.7 μg/mL, indicating mild-to-moderate cytotoxicity toward the tested cell line, as shown in Figure 7a.

**FIGURE 8:**
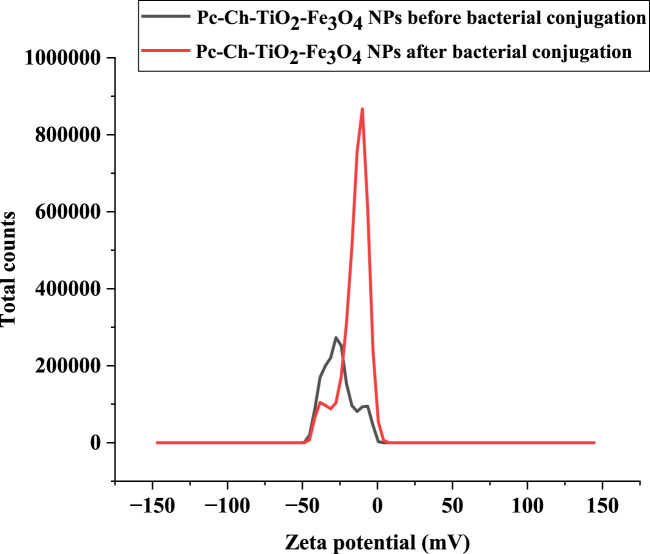
Zeta potential measurement of Pc–Ch–TiO_2_–Fe_3_O_4_ NPs before and after bacterial conjugation, suspended in deionised water.

Furthermore, phycocyanin, a natural pigment-protein rich in amino acids, was conjugated with Ch–TiO_2_–Fe_3_O_4_ nanoparticles, which may contribute to reduced cytotoxic effects toward Vero cells. In addition, Figure 7b illustrates a gradual increase in anti-proliferative effects on the Vero cell line with increasing nanoparticle concentration. Overall, these findings indicate that the Pc–Ch–TiO_2_–Fe_3_O_4_ nanoparticles possess favourable biocompatibility and toxicity toward normal cells, supporting their potential for biomedical applications.

### 3.11 Zeta potential

Zeta potential analysis using a Malvern Zetasizer (United Kingdom) revealed a significant alteration in the surface charge characteristics of Pc–Ch–TiO_2_–Fe_3_O_4_ nanoparticles after bacterial conjugation, confirming an electrostatic interaction between the nanoparticles and *Lactobacillus* sp. Before bacterial conjugation, the nanoparticles exhibited a broader negative zeta potential distribution, ranging from approximately −25 mV, indicating moderate colloidal stability. After bacterial conjugation, the zeta potential peak shifted toward less negative values near −14 mV, accompanied by a sharp increase in total counts. This shift indicates neutralisation of surface charges due to electrostatic attraction between the negatively charged bacterial cell wall components and positively charged functional groups present on the nanoparticle surface. The increased peak intensity further confirms successful bacterial attachment and stable nanoparticle–bacterial conjugation through electrostatic interactions.

### 3.12 Cell separation/adhesion using Pc–Ch–TiO_2_–Fe_3_O_4_ NPs in batch experiment and lab fermentor

**FIGURE 9:**
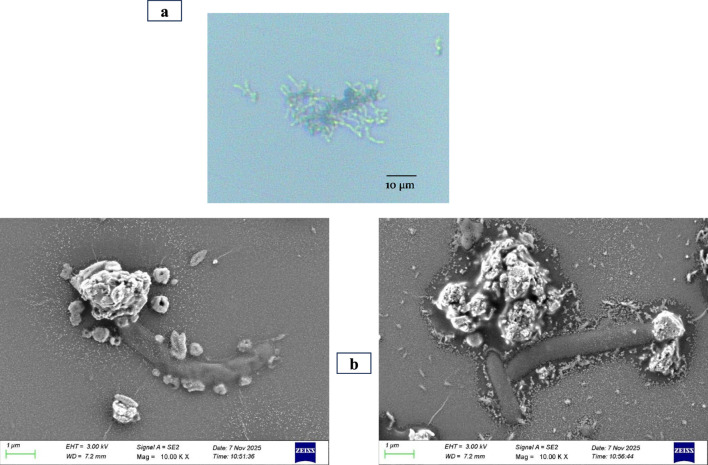
**(a)** Fluorescent microscopic image of Pc–Ch–TiO_2_–Fe_3_O_4_ NPs with *Lactobacillus* sp. **(b)** FESEM image of Pc–Ch–TiO_2_–Fe_3_O_4_ NPs captured by *Lactobacillus* sp.

**FIGURE 10:**
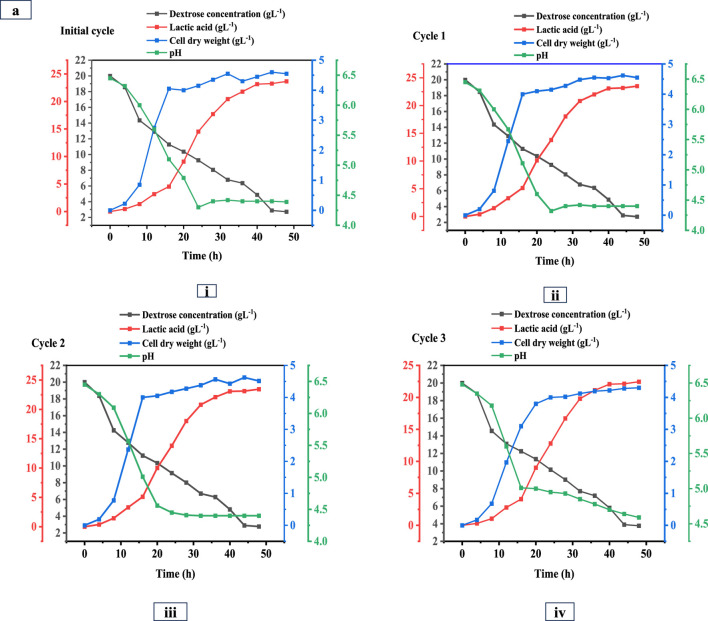
**(a)** Biomass concentration, substrate consumption, lactic acid production, and pH dynamics for four repetitive cycles: (i) initial cycle, (ii) cycle 1, (iii) cycle 2, and (iv) cycle 3.

During the optimisation process of Pc–Ch–TiO_2_–Fe_3_O_4_ NPs at the conical flask level, it was observed that *Lactobacillus* sp. were well-separated at an optimum concentration of 40 mg/100 mL Pc–Ch–TiO_2_–Fe_3_O_4_ NPs, with a separation efficiency of 63.88% ± 15.93%. Meanwhile, at a lower concentration of 10 mg/100 mL, Pc–Ch–TiO_2_–Fe_3_O_4_ NPs showed 41.75% ± 21.17%, and at 50 mg/100 mL, it decreased to 47.88% ± 1.45%. This is represented in Figure 9c. An increase in sample concentration and a decrease in cell separation may be associated with nanoparticle-induced stress on the cell, leading to reduced cell viability. A decrease in cell viability showed the toxicity of the sample at higher concentrations (Figures 9a,b **)**. Control experiments performed without nanoparticles using the conventional centrifugation method yielded a higher bacterial separation efficiency of 88.6% ± 0.46%. Pc–Ch–TiO_2_–Fe_3_O_4_ nanoparticles conjugated with phycocyanin were used for cell recovery through magnetic separation, with an efficiency of approximately 63.88% ± 15.93% at the optimised nanoparticle concentration. Similarly, Ch–TiO_2_–Fe_3_O_4_ nanoparticles without phycocyanin exhibited lower separation efficiency, approximately 28.80% ± 0.36%, than Pc-conjugated nanoparticles, demonstrating the contribution of phycocyanin conjugation toward moderate bacterial adhesion and interaction. Another reason for cell separation efficiency is the agglomeration of nanoparticles with a retained concentration of phycocyanin, as depicted in a fluorescent microscope, LYNX, India, and FESEM analysis (Figures 9a,b). It was observed that the Pc–Ch–TiO_2_–Fe_3_O_4_ cluster was predominantly and directly attached to the bacterial cell wall. Pc–Ch–TiO_2_–Fe_3_O_4_ nanoparticles bind to *Lactobacillus* sp. due to electrostatic interactions shown in Figure 10, hydrogen bonding, and ligand–receptor affinity. Chitosan’s positively charged amino and hydroxyl functional groups on the nanoparticle surface interact electrostatically with negatively charged components of the bacterial cell wall, such as teichoic acids and peptidoglycan layers.

Furthermore, the abundance of hydroxyl, amine, and carboxyl groups in phycocyanin and chitosan promotes hydrogen bond formation with bacterial membrane surface proteins and polysaccharides. Importantly, these associations are non-covalent and reversible, allowing cells to detach under regulated conditions such as pH, ionic strength, or magnetic field exposure. Hence, the present analysis led to an effective conventional method using Pc–Ch–TiO_2_–Fe_3_O_4_ NPs for cell separation. Post-separation cell viability analysis revealed that the majority of bacterial cells remained viable in the centrifugation method, with approximately (2.89 ± 0.05) × 10^9^ CFU/mL. In comparison, Pc–Ch–TiO_2_–Fe_3_O_4_ nanoparticles showed a viability of (2.23 ± 0.2) × 10^9^ CFU/mL, indicating that the optimised nanoparticle concentration exhibited acceptable biocompatibility toward *Lactobacillus*. In Ch–TiO_2_–Fe_3_O_4_ nanoparticles without phycocyanin, cell viability count was found to be (7.8 ± 0.2) × 10^9^ CFU/mL. Using magnetic adsorbents for microbial cell recovery offers significant operational and economic advantages over conventional membrane filtration systems. These benefits arise from reduced membrane fouling, lower energy requirements, decreased dependence on consumable filters, and the ability to recycle magnetic particles for repeated use in downstream processing applications. The consistency of the Pc–Ch–TiO_2_–Fe_3_O_4_ NPs was again tested in a lab fermentor of 5-L capacity. Here, along with cell separation efforts, efforts were made to detect cell regrowth for lactic acid production. Biomass concentration, cell dry weight, substrate concentration, lactic acid production, and pH were evaluated at various time intervals.

### 3.13 Growth kinetics evaluation

The growth behaviour and metabolite production of *Lactobacillus* sp. were evaluated over four batch cycles, each with a 48-h incubation period. Across all cycles, a lag phase of approximately 5 h was observed, followed by an exponential growth phase ranging from 12 to 30 h. Supplementary Table S1 summarises the trends in biomass accumulation and dextrose utilisation for each cycle. As shown in (Figure 10a, i), the highest cell dry weight (CDW) achieved was 4.55 ± 0.06 g L^-1^, accompanied by a maximum lactic acid concentration of 23.65 ± 0.24 g L^-1^ in 48 h. In the initial cycle (without the addition of Pc–Ch–TiO_2_–Fe_3_O_4_ NPs), the culture pH declined to 4.39 ± 0.01. During the exponential phase (0 h–18 h), CDW increased from 0.85 ± 0.03 to 4.55 ± 0.06 g L^-1^, indicating active biomass generation. Cycle 1 (in addition to 400 mg/L of Pc–Ch–TiO_2_–Fe_3_O_4_ NPs) (Figure 10a, ii) demonstrated similar growth characteristics, with CDW reaching 4.48 ± 0.09 g L^-1^ after 32 h. The lactic acid concentration peaked at 23.5 ± 0.23 g L^-1^, with a significant yield attributed to the consumption of dextrose, which decreased from 20.0 ± 0.00 g L^-1^ to 2.73 ± 0.04 g L^-1^ after 48 h. Biomass increased from 0.2 ± 0.01 to 4.0 ± 0.09 g L^-1^ within 16 h during the exponential growth phase. Cycle 2 exhibited a shorter exponential phase, terminating at 8 h, with a corresponding CDW of 4.05 ± 0.08 g L^-1^ (Figure 10a, iii). This biomass level remained constant for the next 20 h, followed by a slight increase to 4.515 ± 0.06 g L^-1^ at the end of incubation (48 h). Biomass productivity was calculated as 0.072 g L^-1^ h^-1^. The lactic acid concentration reached 23.45 ± 0.21 g L^-1^, and the yield was associated with a dextrose consumption of 2.77 ± 0.05 g L^-1^ over 48 h.

In contrast, cycle 3 (Figure 10a, iv) showed a delayed onset of exponential growth, beginning at 12 h. At this stage, CDW reached 3.8 ± 0.08 g L^-1^. The biomass productivity for this cycle was 0.069 g L^-1^ h^-1^. Lactic acid production occurred during both exponential and stationary phases, with a final concentration of 22.45 ± 0.22 g L^-1^, corresponding to the consumption of 16.23 g L^-1^ of dextrose. Hence, it was observed that lactic acid production was not affected in cycles 1 and 2 during cell regrowth, while it decreased at the end of cycle 3. This decline was mainly due to a decrease in cell separation efficiency from 71% (cycle 1) to 65% (cycle 3), along with the lack of phycocyanin, which is present around the nanoparticles. The comparative analysis of four cycles revealed the potential of Pc–Ch–TiO_2_–Fe_3_O_4_ NPs for cell addition, cell regrowth behaviour, and metabolic activity of *Lactobacillus* sp. under identical culture conditions. Despite minor variations in the timing and extent of exponential growth phases, all cycles demonstrated efficient biomass accumulation and lactic acid production, with final concentrations exceeding 22 g L^-1^. The highest biomass yield (4.55 ± 0.06 g L^-1^) and lactic acid concentration (23.65 ± 0.24 g L^-1^) were achieved in the initial cycle. Notably, cycle 2 exhibited the highest biomass productivity (0.072 g L^-1^ h^-1^), while cycle 3 reflected delayed growth initiation yet maintained significant metabolite output. Experimental data for initial biomass concentration (X_0_) between 0.17 and 0.2 g L^-1^ and initial lag phase (t_0_) time delay at 5 h in each cycle. These findings confirm the suitability of the method for *Lactobacillus* cultivation and highlight the strain’s robust fermentative performance across repeated batch operations. The results also provide valuable baseline data for scaling up bioprocesses aimed at lactic acid production using Pc–Ch–TiO_2_–Fe_3_O_4_ NPs. This new technique, using Pc–Ch–TiO_2_–Fe_3_O_4_ NPs, is not only used for cell separation but is also, perhaps, used simultaneously to increase the purity of lactic acid in downstream processing. Hence, this technique can be implemented in industries to avoid the need for a cell-membrane filtration unit.

### 3.14 Kinetic growth model

#### 3.14.1 Contois model

**FIGURE 11:**
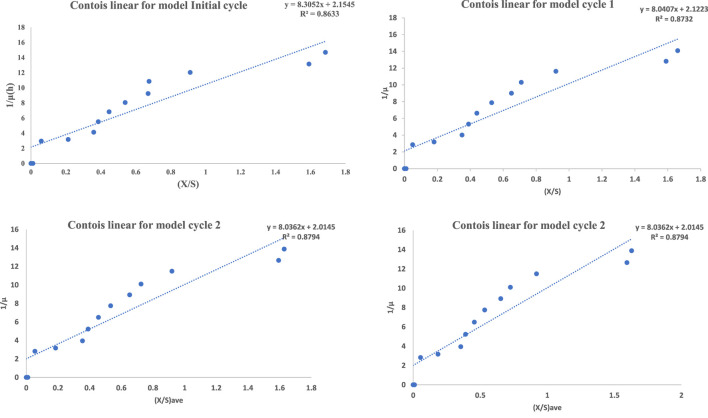
Linearised form of the Contois kinetic model was applied to fit the experimental data on substrate utilisation and cell growth for the four studied cycle *Lactobacillus* sp. strains cultivated in a lab fermenter.

Table 1 and Figure 11 present the obtained µ_max_ (h^-1^), K_s_, and R^2^ values. The model exhibited a strong fit to the experimental data, with R^2^ values ranging from 0.8633 to 0.9303, demonstrating reliable predictive performance of the Contois model under the analysed conditions. A progressive increase in µ_max_, from 0.464 h^-1^ in the initial cycle to 0.651 h^-1^ in cycle 3, indicates enhanced metabolic activity and effective physiological adaptation of the culture over successive batches. The K_s_ values (3.788 g L^-1^–7.53 g L^-1^) remained within an acceptable range, indicating stable substrate affinity throughout the fermentation cycles. However, the elevated K_s_ in cycle 3 may reflect increased substrate demand associated with higher biomass density. Overall, these findings confirm that the Contois model accurately describes the growth behaviour of *Lactobacillus* sp. in repeated-batch operation. The improvement in kinetic parameters further supports the effectiveness of the nanoparticle-assisted cell-retention system in sustaining consistent fermentation performance.

#### 3.14.2 Monod model

**FIGURE 12:**
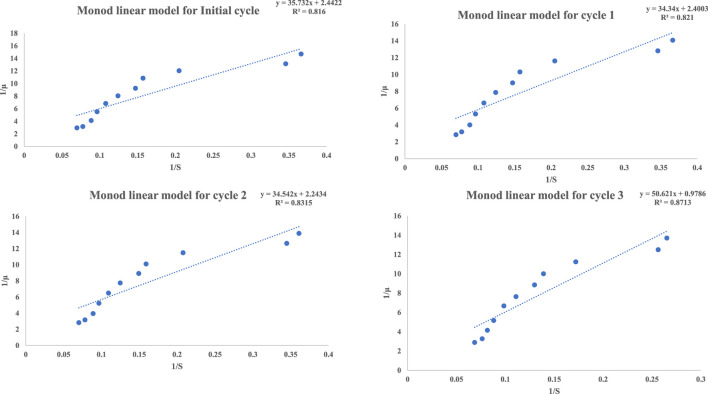
Linearised form of the Monod kinetic model was applied to fit the experimental data for the four studied cycle *Lactobacillus* sp. strains cultivated in a lab fermenter.

Figure 12 shows moderate agreement between the Monod model and experimental data, with R^2^ values ranging from 0.816 to 0.8713. The maximum specific growth rate (µmax) gradually increased from 0.401 h^-1^ in the initial cycle to 1.05 h^-1^ in cycle 3, showing better microbial adaptability and growth activity during repeated-batch operation. Similarly, Ks values increased from 14.63 to 51.73 g L^-1^, showing a gradual loss in substrate affinity at later cycles. This could be due to increased biomass concentration, substrate competition, or physiological stress under repetitive fermentation conditions. The Monod model accurately described substrate-limited growth, but its lower R^2^ values indicate that substrate utilisation alone cannot fully explain the system’s complex growth characteristics.

#### 3.14.3 Logistic model

**FIGURE 13:**
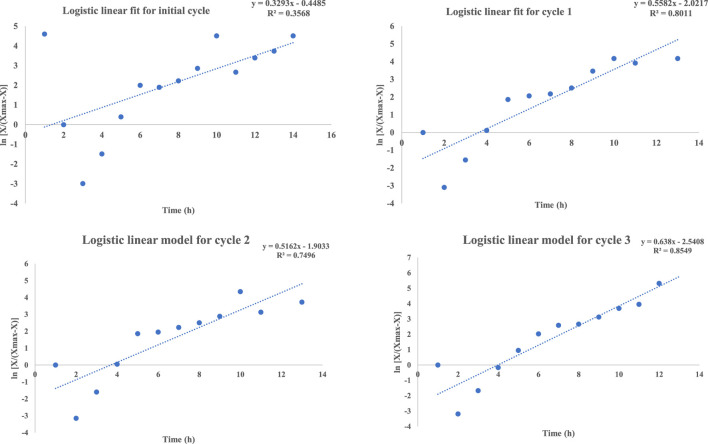
Linearised form of the logistic kinetic model was applied to fit the experimental data for the four studied cycle *Lactobacillus* sp. strains cultivated in a lab fermenter.

The logistic model was utilized to examine using Equation 13 biomass growth behaviour irrespective of substrate limitation, as shown in Figure 13. Cycles 1–3 showed significantly better fitting than the initial cycle, with R^2^ values ranging from 0.3568 to 0.8549. The increase in µmax from 0.3293 h^-1^ to 0.638 h^-1^ indicates increased cell proliferation throughout subsequent fermentation cycles. However, the comparatively poor fitting in the initial cycle indicates that the logistic model may fail to describe the culture’s early adaptation phase appropriately. The improved fitting in the later cycles could be due to stable growth conditions and bacterial cell adaptation to the nanoparticle-assisted separation technology.

#### 3.14.4 Gompertz model

**FIGURE 14:**
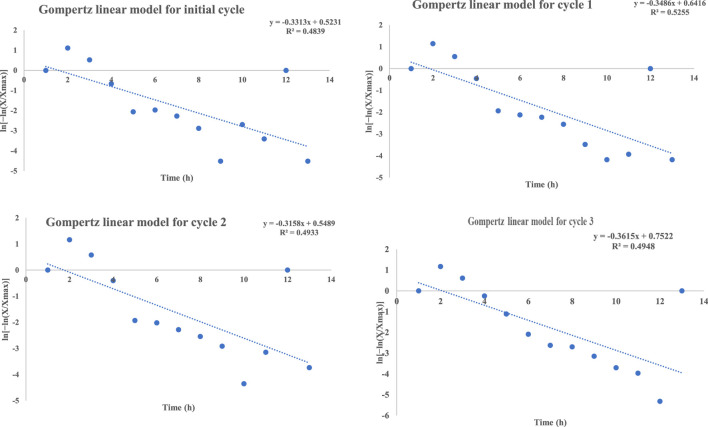
Linearised form of the Gompertz kinetic model was applied to fit the experimental data for the four studied cycle *Lactobacillus* sp. strains cultivated in a lab fermenter.

The Gompertz model was plotted using Equation 15, did not fit the data very well. It had R^2^ values ranging from 0.4839 to 0.5255. This can be seen in Figure 14. The Gompertz model showed consistent maximum growth rates. These rates ranged from 0.3158 to 0.3615 h^-1^. This shows that the microbes’ growth was fairly stable throughout fermentation. The Gompertz model is used to describe the lag phase and sigmoidal growth of microbes. In this study, the model did not fit the data very well. This might be because the fermentation system lacked a lag phase when run in repeated batches. The Gompertz model is usually good for this kind of thing. It also fits well with the experimental data from the fermentation system.

The Contois model outperformed the Monod, logistic, and Gompertz models, with higher R^2^ values (0.8633–0.9303). The Contois model’s superior predictive accuracy indicates that microbial growth in the current system was influenced by both substrate concentration and biomass density, as is typical of high-cell-density fermentation systems. As a result, the Contois model was deemed more adequate for describing *Lactobacillus* sp.'s repeated-batch fermentation behaviour in nanoparticle-assisted cell separation.

### 3.15 HPLC analysis for lactic acid

**FIGURE 15:**
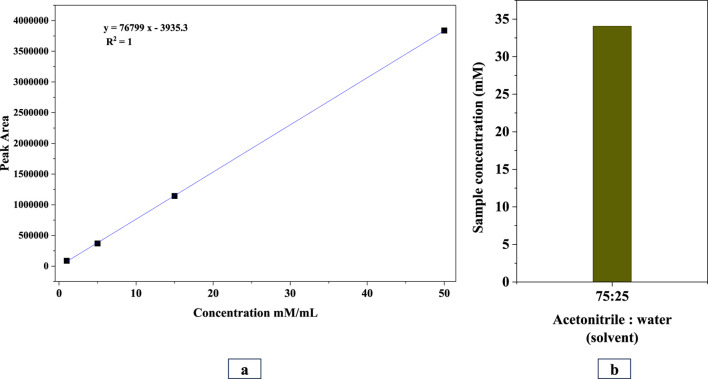
**(a)** Calibration curve for L-Lactic acid in the linear range from 1 to 50 mM/mL. **(b)** Quantification of L-Lactic acid with a 75:25 ratio of solvent.

HPLC analysis was performed to evaluate the lactic acid purity after selective bacterial separation, and the results confirmed enhanced purification shown in Figures 15a, b. A Shimadzu DGU-20A5R (Japan) reverse-phase C18 column HPLC system equipped with a UV/Vis detector and manual sample injector was utilized for the analysis. Chromatographic separation was performed using an analytical column (250 mm × 4.6 mm, 5 µm particle size). The column temperature was maintained at 40 °C ± 2 °C, and the mobile phase was delivered at 1.0 mL/min under isocratic conditions. The mobile phase consisted of solvent A (3.1 × 10^−2^ mol/L H_3_PO_4_ in water) and solvent B (HPLC-grade acetonitrile) in a ratio of 25:75 (v/v). The injection volume was fixed at 20 µL, and detection was monitored at 210 nm.

Standard lactic acid solutions (1, 5, 10, and 50 mM) were prepared using HPLC-grade acetonitrile and filtered through a 0.45-µm membrane filter before analysis. Similarly, all samples and solvents were filtered before injection. The retention time of lactic acid was determined to be approximately 6.343 min, and it was quantified successfully. The developed HPLC method demonstrated good reproducibility, repeatability, and effective separation performance. The concentration of lactic acid obtained from the analysis was 34.0485 mM/mL. These findings confirmed the suitability of the developed chromatographic method for accurate separation and quantification of lactic acid. Supplementary Figure S1 represents the real-time sample run data. Overall, the HPLC results demonstrate the successful detection and quantification of lactic acid after selective bacterial isolation. The new chromatographic method demonstrated good reproducibility and repeatability, with efficient separation, indicating its suitability for lactic acid analysis.

## 4 Discussion

The produced Pc–Ch–TiO_2_–Fe_3_O_4_ nanoparticles were successfully characterised and analysed for their structural, physicochemical, and biological properties. UV–Visible spectroscopy showed the presence of phycocyanin, with a distinctive absorption peak at 620 nm, confirming successful extraction and conjugation. XRD study showed distinct diffraction peaks for Fe_3_O_4_ and TiO_2_ phases, indicating the nanocomposite’s crystalline character without structural distortion following functionalisation. FT-IR spectra revealed the presence of important functional groups such as O–H, N–H, and C–H stretching vibrations, along with amide bands, confirming the successful coating of chitosan and phycocyanin on the nanoparticle surface. The presence of Fe–O and Ti–O bands provided more evidence that the hybrid nanostructure had formed. VSM study revealed superparamagnetic behaviour with adequate magnetic responsiveness for effective separation. FESEM images revealed agglomerated spherical nanoparticles, confirming nanoscale morphology. The TGA findings showed low weight loss, showing that the nanocomposite is thermally stable. XPS analysis revealed the presence of Fe, Ti, O, and organic functional groups, indicating surface modification. Cytotoxicity analysis found little toxicity at lower dosages, validating biocompatibility. Furthermore, cell separation assays showed that these nanoparticles can effectively bind and recover bacterial cells, emphasizing their potential for microbial separation.

This study evaluates the physicochemical properties and biological performance of Pc–Ch–TiO_2_–Fe_3_O_4_ nanoparticles. The findings are supported by extensive comparisons to previous findings, validating their structural integrity, functionalisation, and applicability in bioprocess systems. In UV–Visible spectroscopy, phycocyanin extraction and purification have been widely refined utilising multiple techniques, with freeze–thaw extraction paired with ammonium sulphate precipitation yielding approximately 13.20 mg/mL (Fratelli et al., 2022), while refined methods incorporating potassium buffer conditions and ultrasonication obtained much greater yields of 44.24 mg/g biomass, albeit with intermediate purity indices (Gorgich et al., 2020). The characteristic absorption peak at approximately 620 nm remains a definitive indicator of phycocyanin presence and purity, with higher absorbance values correlating with increased pigment concentration and structural integrity (Abd El-Baky and El-Baroty, 2012). Additionally, the detection of secondary peaks in the 320 nm–350 nm range associated with phycobilin chromophores and carotenoids, which are commonly observed in crude extracts of Spirulina-derived phycocyanin, reflects the coexistence of accessory pigments (Morançais et al., 2018). These spectral features collectively confirm the successful extraction and retention of functional chromophores, which are essential for subsequent nanoparticle conjugation.

X-ray diffraction analysis contributes to the creation of a well-defined crystalline nanocomposite structure. The observed diffraction patterns are compatible with previously reported standard peaks of the Fe_3_O_4_ and TiO_2_ phases, along with the ilmenite-phase FeTiO_3_, which exhibits characteristic reflections at specific θ values owing to its rhombohedral lattice structure (Ali et al., 2025). Similarly, pure magnetic nanoparticles and their variants exhibit diffraction peaks from planes such as 220, 311, 400, 511, and 440, confirming the crystalline integrity of the magnetic core (Muthuvelu et al., 2020). The preservation of these diffraction peaks with no significant shifts indicates that surface functionalisation with chitosan and phycocyanin does not alter the core crystal structure but rather forms a stable coating layer that maintains the magnetic properties while allowing biological interactions.

FT-IR study provided additional insights into surface chemistry and functional group interactions, revealing distinctive absorption bands associated with the hydroxyl, amine, and aliphatic functional groups. Organic components such as chitosan and phycocyanin are confirmed by the existence of O–H and N–H stretching vibrations at approximately 3,400 cm^-1^, along with C–H stretching at approximately 2,900 cm^-1^ (Nair et al., 2023; Morançais et al., 2018, Kurniawan et al., 2025). In this literature, 3,413 cm^-1^ was assigned to O–H stretching, 2,929 cm^-1^ to C–H stretching, and 1,458 cm^-1^ to CH_2_ (Mano et al., 2003). The presence of amide bands at approximately 1,650 cm^-1^ confirms the effective conjugation of phycocyanin onto the nanoparticle surface and aligns with the reported amide I and II vibrations of pure chitosan (improvement in ductility of chitosan) (Kolhe and Rangaramanujam, 2003). Additional peaks in the lower wavenumber range, particularly at approximately 590 cm^-1^, correspond to Ti–O bonding. Literature values at approximately 595 and 443 cm^-1^ further indicate the presence of metal–oxygen couplings (Rajput et al., 2016; Grahn et al., 1998). These functional groups not only validate successful synthesis but also enable nanoparticle–cell interactions through electrostatic attraction, hydrogen bonding, and ligand–receptor interactions.

Vibrating sample magnetometry (VSM) reveals magnetic behaviour similar to that of previously described ferrimagnetic and superparamagnetic nanoparticles. Saturation magnetisation values for similar systems range from 40.32 to 84.29 emu/g, depending on the composition and morphology (Hingrajiya and Patel, 2023). In comparison, TiO_2_- or SiO_2_-coated Fe_3_O_4_ nanoparticles typically exhibit values between 40 and 70 emu/g, which is indicative of superparamagnetic behaviour suitable for magnetic separation applications (Herbei et al., 2025). In contrast, complex nanocomposites such as Fe_3_O_4_/TiO_2_/CoMoO_4_ have lower magnetisation values (11 emu/g–30 emu/g) due to higher non-magnetic content (Anandan et al., 2017). These studies indicate that the produced nanoparticles retain adequate magnetic responsiveness when functional coatings are added, enabling effective recovery and reuse.

Morphological examination by SEM reveals the formation of agglomerated spherical nanoparticles that are consistent with green-synthesised Fe_3_O_4_ systems that exhibit lustre formation due to magnetic interactions (Mossa Mishbak et al., 2025). Fe-doped TiO_2_ structures contain flower-like morphologies mimicking edelweiss, showing significant particle–particle interactions and surface complexity. The presence of distinctive elemental signals at energy levels corresponding to iron validates the composition (Gareso et al., 2025). In the reported literature, a TGA analysis of thermal stability indicates low mass loss, comparable to that of unfunctionalised iron oxide nanoparticles, with approximately 1.8% weight loss under inert conditions (Kunc et al., 2022), highlighting the stability of the inorganic core and the controlled degradation of organic components.

Surface chemical composition and oxidation states, as described in XPS studies of comparable nanomaterials, provide additional support for the composite structure. Fe 2p peaks at 710 and 724 eV indicate the presence of Fe^2+^ and Fe^3+^ states (Anandan et al., 2017), while O 1s peaks at approximately 530 eV–531 eV indicate oxide species (Ahmad et al., 2025). Shifts in oxygen binding energies, as observed in biosynthesised nanoparticles, further indicate interactions with adsorbed species (Shehata et al., 2026). Ti 2p signals at approximately 458.5 eV confirm TiO_2_ incorporation (Hajareh Haghighi et al., 2024), and nitrogen-related peaks between 398 and 401 eV correspond to amine functionalities from organic coatings (Corsini et al., 2025). Additionally, carbon-related peaks (C–C, C–O, and C=O) indicate successful coating and composite formation (Moss et al., 2026; Schwegmann et al., 2010).

However, synthesized Ag NPs-*T. suecica* at 1.5:3 ratio revealed low cytotoxicity with IC_50_ 38.01- 45.7 µg/mL^-1^ against MCF-7 and non-cytotoxic against Vero cells (Hussein et al., 2020). Magnetic nanoparticle (MNP)-based aggregation assays have been developed for the in vitro detection of clinically significant biomolecules (Mancilla et al., 2025). In other hand the particular literature reported that amino acids 1 and 4 shows less cytotoxicity towards Vero cell line (Mazurenko et al., 2025). In comparison, iron oxide nanoparticles sorbed by bacteria exhibit a negative surface charge*. E. coli* has a higher negative zeta potential (−50 mV) than *S. cerevisiae* (−5 mV) (Schwegmann et al., 2010). Notably, functionalised nanoparticles containing phycocyanin and chitosan exhibit strong binding affinity for bacterial cells, leading to high separation efficiency, including total capture in some systems (Nair et al., 2023; Huy et al., 2014; Mulo et al., 2020). The addition of functional groups, such as amino and hydroxyl moieties, enhances the electrostatic and hydrogen-bonding interactions with bacterial cell walls, enabling selective and efficient separation. Furthermore, the reusability of such systems has been established via repeated adsorption–desorption cycles with excellent efficiency retention (Hingrajiya and Patel, 2023).

Microbial growth kinetics studies further highlight the influence of environmental conditions and supplementation strategies on biomass production and growth rates. *Lactobacillus paracasei* inactivated cells were added to *Saccharomyces cerevisiae* as a para-probiotic supplement to increase cell viability during biomass storage at different concentrations. Biomass yield of approximately 5.078 g/L was achieved in 48 h with 5 % para-probiotic supplement (Pihurov et al., 2025). In this literature, *Lactobacillus acidophilus* was cultured in MRS and single source of carbon (SSC) media. Biomass concentration reached approximately 1.3 g/L during the high exponential phase, and SSCM cultivation media reached approximately 2.5 g/L during the low exponential phase (Isopencu et al., 2025).

The authors reported that µmax increased progressively with increasing paraprobiotic concentrations, reaching the highest value in the 15% supplementation treatment, demonstrating that non-viable LAB components accelerated yeast cell division and shortened the generation time during exponential growth. Model fitting was evaluated using R^2^ values ranging from 0.538 to 0.752, indicating moderate predictive capability given the biological complexity of yeast–para-probiotic interactions (Pihurov et al., 2025). In another study, different types of *Lactobacillus* strains were cultured in semi-solid fermentation (SSF) medium and MRS medium for 30 h. Higher cell density was observed in the SSF medium than with the MRS medium, including *Lb. rhamnosu*s, *Lb. paracasei*, *Lb. reuteri*, *Lb. plantarum*, and *Lb. pentosus*, which shows µ_max_ (h^-1^) calculated at approximately 0.21, 0.27, 0.31, 0.22, and 0.37, respectively, in the SSF medium at 30 h. The MRS medium revealed that *Lb. rhamnosu*s, *Lb. paracasei*, Lb. *reuteri*, *Lb. plantarum*, and *Lb. pentosus* shows µ_max_ (h^-1^) at approximately 0.12, 0.18, 0.14, 0.15, and 0.23, respectively, in 30 h (Śliżewska et al., 2020). The Monod kinetic model is used to describe the specific growth rate of the bacterial strain (Mulo et al., 2020), whereas the logistic and Gompertz models are applied to analyse total bacterial counts as they capture multiple growth-related parameters (Akın et al., 2020). Overall, the collective evidence from the structural, thermal, magnetic, surface, and biological analyses strongly confirms that Pc–Ch–TiO_2_–Fe_3_O_4_ nanoparticles possess a synergistic combination of stability, functionality, and biocompatibility, making them highly suitable for efficient microbial cell separation and advanced bioprocess applications, which is in strong agreement with previously reported studies. HPLC analysis was carried out using a reverse-phase C18 column, with solvent A: water with phosphoric acid, solvent B: acetonitrile (88:12), flow rate: 1 mL/min, and sample injection volume: 20 µL. The results showed that PLA polymers contained 0.500 mg/mL–2.500 mg/mL at a retention time of approximately 4.9 min.

## 5 Conclusion

To conclude, an innovative application of Pc–Ch–TiO_2_–Fe_3_O_4_ NPs for cell separation offers significant potential to reduce both upstream and downstream processes for lactic acid production. Analytical characterisation was carried out, and analyses of the morphology, elemental composition, functional groups, elemental state, and magnetic energy confirmed the properties of the synthesised nanoparticles. A biocompatibility assay for nanoparticles revealed that Pc–Ch–TiO_2_–Fe_3_O_4_ NPs are non-toxic to viable cells at lower concentrations. Cell separation was confirmed using a fluorescent microscope and FESEM. Overall, novel findings highlight the potential of Pc–Ch–TiO_2_–Fe_3_O_4_ nanoparticles as an effective, reusable, and biocompatible platform for magnetic cell separation. This approach offers a promising industrial fermentation by enabling selective cell recovery, retention, and reuse. The demonstrated structural stability, magnetic responsiveness, and biological compatibility of the nanoparticle position it as a valuable candidate for future applications in microbial bioprocessing, biomedical separations, and other magnetically driven biotechnological operations.

## Data Availability

The original contributions presented in the study are included in the article/Supplementary Material, further enquiries can be directed to the corresponding authors.
