# Supplementary material for: Construction of novel phycocyanin conjugated chitosan–TiO2–Fe3O4 nanoparticles for Lactobacillus retainment and application in cyclic batch fermentation
Source: Front Bioeng Biotechnol. 2026 Aug 5;14:1856231. doi: 10.3389/fbioe.2026.1856231 (PMC13486296; doi:10.3389/fbioe.2026.1856231)
Supplement: Supplementary file 1 [file Supplementaryfile1.docx]

**Supplementary material**

**Table 1 (S1):** Biomass concentration, substrate concentration, lactic acid concentration, specific growth rate and pH for four studied cycles of *Lactobacillus* spp. in a lab fermenter at 37 ̊ C with 50 rpm speed

| **Time (h)** | **Cycles** | **X (g L^-1^)**  **Mean ± SD** | **S (g L^-1^)**  **Mean ± SD** | **LA (g L^-1^)**  **Mean ± SD** | **μ (h^-1^)**  **Mean ± SD** | **pH**  **Mean ± SD** |
| --- | --- | --- | --- | --- | --- | --- |
| 0 | Initial cycle  Cycle 1  Cycle 2  Cycle 3 | 0.00 ± 0.00  0.00 ± 0.00  0.00 ± 0.00  0.00 ± 0.00 | 20.00 ± 0.00  20.00 ± 0.00  20.00 ± 0.00  20.00 ± 0.00 | 0.00 ± 0.00  0.00 ± 0.00  0.00 ± 0.00  0.00 ± 0.00 | 0.000 ± 0.000  0.000 ± 0.000  0.000 ± 0.000  0.000 ± 0.000 | 6.45 ± 0.00  6.45 ± 0.00  6.45 ± 0.00  6.45 ± 0.00 |
| 4 | Initial cycle  Cycle 1  Cycle 2  Cycle 3 | 0.22 ± 0.01  0.20 ± 0.01  0.19 ± 0.01  0.17 ± 0.01 | 18.57 ± 0.12  18.48 ± 0.11  18.38 ± 0.12  18.80 ± 0.11 | 0.45 ± 0.03  0.40 ± 0.02  0.37 ± 0.03  0.31 ± 0.02 | 0.000 ± 0.000  0.000 ± 0.000  0.000 ± 0.000  0.000 ± 0.000 | 6.32 ± 0.02  6.31 ± 0.02  6.30 ± 0.02  6.35 ± 0.02 |
| 8 | Initial cycle  Cycle 1  Cycle 2  Cycle 3 | 0.85 ± 0.03  0.81 ± 0.02  0.78 ± 0.03  0.68 ± 0.03 | 14.35 ± 0.15  14.35 ± 0.12  14.25 ± 0.14  14.56 ± 0.13 | 1.36 ± 0.05  1.50 ± 0.04  1.45 ± 0.05  1.05 ± 0.04 | 0.337 ± 0.008  0.349 ± 0.008  0.353 ± 0.009  0.346 ± 0.008 | 6.00 ± 0.03  6.00 ± 0.03  6.09 ± 0.03  6.18 ± 0.03 |
| 12 | Initial cycle  Cycle 1  Cycle 2  Cycle 3 | 2.75 ± 0.08  2.45 ± 0.07  2.37 ± 0.08  1.97 ± 0.07 | 12.90 ± 0.11  12.90 ± 0.10  12.79 ± 0.11  13.09 ± 0.10 | 3.18 ± 0.09  3.30 ± 0.08  3.30 ± 0.08  2.80 ± 0.07 | 0.315 ± 0.007  0.313 ± 0.007  0.315 ± 0.007  0.306 ± 0.007 | 5.60 ± 0.03  5.67 ± 0.03  5.57 ± 0.03  5.60 ± 0.03 |
| 16 | Initial cycle  Cycle 1  Cycle 2  Cycle 3 | 4.05 ± 0.10  4.00 ± 0.09  4.00 ± 0.10  3.10 ± 0.09 | 11.28 ± 0.14  11.28 ± 0.11  11.24 ± 0.13  12.24 ± 0.12 | 4.54 ± 0.12  5.14 ± 0.10  5.10 ± 0.11  4.10 ± 0.09 | 0.242 ± 0.006  0.249 ± 0.006  0.253 ± 0.006  0.241 ± 0.006 | 5.10 ± 0.02  5.11 ± 0.02  5.01 ± 0.02  5.01 ± 0.02 |
| 20 | Initial cycle  Cycle 1  Cycle 2  Cycle 3 | 4.00 ± 0.09  4.10 ± 0.08  4.05 ± 0.08  3.80 ± 0.08 | 10.37 ± 0.10  10.37 ± 0.10  10.35 ± 0.10  11.35 ± 0.10 | 9.08 ± 0.20  10.10 ± 0.22  10.01 ± 0.20  9.01 ± 0.18 | 0.181 ± 0.005  0.181 ± 0.005  0.191 ± 0.005  0.194 ± 0.005 | 4.79 ± 0.02  4.60 ± 0.02  4.56 ± 0.02  5.00 ± 0.02 |
| 24 | Initial cycle  Cycle 1  Cycle 2  Cycle 3 | 4.15 ± 0.07  4.15 ± 0.08  4.17 ± 0.07  4.00 ± 0.07 | 9.27 ± 0.13  9.27 ± 0.11  9.15 ± 0.12  10.15 ± 0.11 | 14.52 ± 0.31  13.80 ± 0.26  13.80 ± 0.24  12.80 ± 0.22 | 0.146 ± 0.004  0.151 ± 0.004  0.154 ± 0.004  0.157 ± 0.004 | 4.30 ± 0.02  4.32 ± 0.02  4.45 ± 0.02  4.95 ± 0.02 |
| 28 | Initial cycle  Cycle 1  Cycle 2  Cycle 3 | 4.35 ± 0.11  4.27 ± 0.10  4.27 ± 0.09  4.02 ± 0.08 | 8.06 ± 0.10  8.06 ± 0.09  8.01 ± 0.10  9.01 ± 0.09 | 17.70 ± 0.28  18.03 ± 0.29  18.01 ± 0.28  16.71 ± 0.26 | 0.124 ± 0.004  0.127 ± 0.004  0.129 ± 0.004  0.131 ± 0.004 | 4.40 ± 0.01  4.40 ± 0.01  4.41 ± 0.01  4.93 ± 0.01 |
| 32 | Initial cycle  Cycle 1  Cycle 2  Cycle 3 | 4.55 ± 0.09  4.48 ± 0.09  4.38 ± 0.08  4.12 ± 0.08 | 6.79 ± 0.08  6.79 ± 0.08  6.70 ± 0.08  7.70 ± 0.08 | 20.43 ± 0.35  20.82 ± 0.33  20.80 ± 0.30  19.80 ± 0.29 | 0.108 ± 0.003  0.111 ± 0.003  0.112 ± 0.003  0.113 ± 0.003 | 4.42 ± 0.01  4.42 ± 0.01  4.40 ± 0.01  4.85 ± 0.01 |
| 36 | Initial cycle  Cycle 1  Cycle 2  Cycle 3 | 4.30 ± 0.10  4.55 ± 0.08  4.56 ± 0.07  4.19 ± 0.07 | 6.36 ± 0.07  6.36 ± 0.06  6.29 ± 0.06  7.19 ± 0.07 | 21.79 ± 0.32  22.00 ± 0.31  22.10 ± 0.32  21.11 ± 0.30 | 0.092 ± 0.003  0.097 ± 0.003  0.099 ± 0.003  0.1 ± 0.003 | 4.40 ± 0.01  4.40 ± 0.01  4.40 ± 0.01  4.78 ± 0.01 |
| 40 | Initial cycle  Cycle 1  Cycle 2  Cycle 3 | 4.45 ± 0.08  4.53 ± 0.07  4.43 ± 0.08  4.22 ± 0.06 | 4.88 ± 0.09  4.88 ± 0.08  4.81 ± 0.07  5.81 ± 0.06 | 23.15 ± 0.26  23.08 ± 0.24  23.07 ± 0.23  22.07 ± 0.21 | 0.083 ± 0.002  0.086 ± 0.002  0.087 ± 0.002  0.089 ± 0.002 | 4.40 ± 0.01  4.40 ± 0.01  4.40 ± 0.01  4.70 ± 0.01 |
| 44 | Initial cycle  Cycle 1  Cycle 2  Cycle 3 | 4.60 ± 0.07  4.62 ± 0.05  4.62 ± 0.05  4.28 ± 0.05 | 2.89 ± 0.05  2.89 ± 0.05  2.90 ± 0.05  3.90 ± 0.05 | 23.24 ± 0.21  23.17 ± 0.22  23.12 ± 0.20  22.12 ± 0.20 | 0.076 ± 0.002  0.078 ± 0.002  0.079 ± 0.002  0.080 ± 0.002 | 4.40 ± 0.01  4.40 ± 0.01  4.40 ± 0.01  4.64 ± 0.01 |
| 48 | Initial cycle  Cycle 1  Cycle 2  Cycle 3 | 4.55 ± 0.06  4.55 ± 0.06  4.51 ± 0.06  4.30 ± 0.05 | 2.73 ± 0.04  2.73 ± 0.04  2.77 ± 0.05  3.77 ± 0.05 | 23.65 ± 0.24  23.50 ± 0.23  23.45 ± 0.21  22.45 ± 0.22 | 0.068 ± 0.002  0.071 ± 0.002  0.072 ± 0.002  0.073 ± 0.002 | 4.39 ± 0.01  4.40 ± 0.01  4.40 ± 0.01  4.59 ± 0.01 |

**Figure 1 (S1):** Raw data during HPLC analysis
